# Large language models enhance diagnostic reasoning of medical students in rheumatology: a randomized controlled trial

**DOI:** 10.1186/s12909-026-09079-w

**Published:** 2026-03-25

**Authors:** Anna Roemer, Nadine Schlicker, Anna Kernder, Benedikt Albe, Juliana Hack, Martin Hirsch, Andreas Mayr, Sebastian Kuhn, Johannes Knitza

**Affiliations:** 1 School of Medicine, Institute for Digital Medicine, Philipps-Universität Marburg, Baldingerstrasse 1, Marburg, 35043 Germany; 2 School of Medicine, Institute for Artificial Intelligence in Medicine, Philipps-Universität Marburg, Marburg, Germany; 3 Ruhr-University Bochum, Rheumazentrum Ruhrgebiet, Herne, Germany; 4 School of Medicine, Center for Orthopaedics and Trauma Surgery, Philipps-Universität Marburg, Marburg, Germany; 5 Institute for Medical Biometry and Statistics, Philipps-Universität Marburg, Marburg, Germany

**Keywords:** Large language models, Artificial intelligence, EHealth, Clinical decision support systems, Rheumatology, ChatGPT, GPT

## Abstract

**Background:**

Diagnostic errors and delays are common in rheumatology, driven by overlapping symptoms and the rarity of many diseases. While traditional diagnostic decision support systems (DDSS) have seen limited adoption because of high input burden and low perceived value, large language models (LLMs) now offer genuine dialogue and reduced effort, with rapidly improving diagnostic performance, yet empirical evidence on their real-world effectiveness and educational impact is still scarce.

**Objective:**

The aim of this study was to investigate the impact of an LLM on medical students’ diagnostic performance in rheumatology compared with traditional resources.

**Methods:**

In this randomized controlled trial, medical students solved three rheumatology vignettes. For each case, they provided a main diagnosis with confidence and up to four differential diagnoses. Participants were randomized to use ChatGPT-4o plus traditional resources or traditional resources alone. The primary outcome was the proportion of correct top diagnoses. Secondary outcomes were correctness within the top 5 diagnoses, a cumulative diagnostic score, diagnostic confidence, and completion time.

**Results:**

Sixty-eight students (mean [SD] age 24.8 [2.6] years) were randomized. The LLM group identified the correct top diagnosis more often than controls (77.5% vs. 32.4%), yielding an adjusted odds ratio of 7.0 (95% CI 3.8–14.4; *P*<.001), and also exceeded LLM-only performance (77.5% vs. 71.6%). Cumulative diagnostic scores were higher with LLM support (mean [SD] 12.3 [2.3] vs. 6.7 [3.2]; *P*<.001), as was confidence (7.0 [1.3] vs. 6.1 [1.2]; *P*<.001). Completion time increased in the LLM group (505 [131] s vs. 287 [106] s; *P*<.001).

**Conclusions:**

Medical students using an LLM achieved significantly higher diagnostic accuracy than those using conventional resources. Students assisted by the LLM also outperformed the model alone, highlighting the potential of human-AI collaboration. These findings suggest that LLMs may help improve clinical reasoning in complex fields such as rheumatology. However, these findings should be interpreted cautiously, as larger and more diverse studies are needed to confirm their generalisability.

**Trial registration:**

ClinicalTrials.gov, NCT06748170 registered 27 December 2024.

**Supplementary Information:**

The online version contains supplementary material available at 10.1186/s12909-026-09079-w.

## Introduction

Accurate diagnosis is essential for initiating effective treatment and preventing irreversible accrual of damage. However, diagnostic errors remain common [1], and even experienced physicians frequently overestimate or underestimate their diagnostic accuracy [2]. These challenges are particularly pronounced in rheumatology, where many conditions are rare, present with overlapping symptoms, and frequently lead to prolonged diagnostic delays [3].

Diagnostic decision support systems (DDSS), exemplified early on by the seminal INTERNIST-1 [4], were developed to assist clinicians in generating differential diagnoses, yet their clinical adoption has remained limited [5]. Non-adoption of DDSS is mainly caused by an imbalance of required effort and perceived value [5, 6]. Time-consuming data input often reduces user productivity and consequently hinders adoption [5, 6], while genuine dialogue capabilities, a concept proposed by Schwartz as early as 1970 [7], have remained absent. Large language models (LLMs) enable this envisioned dialogue, processing of multimodal data, promising to drastically reduce the effort necessary to use DDSS. The diagnostic capabilities of LLMs have advanced rapidly and are well documented across a range of clinical domains [8], including rheumatology [9, 10]. LLMs are increasingly adopted by rheumatologists [11], particularly as tools for diagnostic decision support [12]. Notably, a recent large-scale national survey found that most rheumatic patients would welcome their rheumatologists using artifical intelligence for clinical decision support [13]. However, empirical evidence demonstrating the real-world effectiveness of DDSS, including LLM-based systems, remains limited [14, 15].

LLM-based DDSS can facilitate cognitive off-loading and potentially improve clinical decision-making [11, 16]. At the same time, risks such as “deskilling, “never-skilling” and “mis-skilling”, such as automation bias, must be acknowledged [11, 16]. For example, prior studies have shown that students sometimes uncritically accepted incorrect suggestions [6, 17]. These concerns highlight the importance of integrating LLMs into undergraduate medical education [11], and recent studies suggest that students not only value such integration but also particularly benefit from hands-on, case-based teaching [17].

Building on this need, we designed a randomized controlled trial to provide medical students with authentic experience using an LLM for diagnostic reasoning and to generate important new evidence on the effects of LLM usage. Rheumatology was chosen as the target field because it combines high diagnostic complexity with relatively limited curricular exposure for medical students. Accordingly, this randomized controlled trial (AIDRARER) evaluated the impact of LLM-assisted diagnostic reasoning in medical students solving rheumatology vignettes. We hypothesized that LLM usage would result in higher diagnostic accuracy compared to the use of conventional resources alone.

## Methods

### Study design

This was a prospective, open-label, parallel group, randomized controlled trial (AIDRARER). The study was reviewed and deemed exempt from formal approval by the institutional review board of Marburg University, Germany (24–221 ANZ). Informed consent was obtained from all participants prior to enrollment and randomization. The trial was prospectively registered at ClinicalTrials.gov (NCT06748170). No financial incentives were offered for participation. Study implementation was overseen by a designated study coordinator (A.R.). Participants could complete the study either remotely or onsite in a supervised computer laboratory. Outcome data were collected using Google Forms (Google LLC, Mountain View, CA, USA). Participation in the study was voluntary and not embedded within the curricular activities. Medical students at Marburg University were recruited through Digital Medicine seminars and informal peer outreach. Eligibility was restricted to students who had begun internal medicine coursework (at least 5^th^ semester). To enable comparability to a previous randomized controlled trial [6], participants received the same three case vignettes featuring typical symptoms of granulomatosis with polyangiitis, rheumatoid arthritis, and systemic lupus erythematosus, sourced from the public online learning center and the Rheum2Learn section of the American College of Rheumatology. These vignettes were previously translated into German [6]. Vignettes were not presented in a randomized order.

### Randomization

Participants were randomized after providing informed consent. The first participant was randomized on January 7 and the last on March 30, 2025. Participants were randomized in a 1:1 ratio. A computer-based random-number generator with variable block sizes was used. Assignment was carried out by a study team member who had no other role in the trial and was not involved in participant recruitment, data collection, or outcome assessment. Participants were randomly assigned to study groups using sealed, opaque envelopes to ensure allocation concealment; however, blinding of participants was not possible due to the nature of the intervention.

### Control group

Participants in the control group were sequentially presented with clinical vignettes and asked to identify a top diagnosis, indicate their diagnostic confidence, and list up to four additional differential diagnoses for each vignette. They were permitted to use any additional resources except large language models, i.e. books or websites. The supervising study coordinator asked participants which additional resources they intended to use, ensured that only permitted resources were accessed, and documented all of them.

### Intervention group

To establish baseline diagnostic accuracy and confidence, participants in the intervention group initially completed the same diagnostic tasks as the control group, with access to any additional resources except LLMs. Subsequently, they were granted access to the web-based ChatGPT-4o interface along with a standardized initial zero-shot prompt strategy (see Supplementary Material 1) and instructions to input the complete case information. If participants failed to enter the correct prompt or complete information, they were asked to repeat the prompt entry. The LLM was instructed to provide five potential diagnoses, each accompanied by a likelihood estimate and a brief explanation (see Supplementary Material 2). Participants were informed that they could freely pose follow-up questions to the model. After interacting with the LLM, participants were again asked to indicate their top diagnosis along with their diagnostic confidence and up to four differential diagnoses for each clinical vignette. Finally, participants completed a short questionnaire assessing LLM acceptance and usability.

### Outcomes

The primary outcome was the proportion of cases with a correct top diagnosis. Secondary outcomes included: (1) the proportion of cases with a correct diagnosis among the top five suggested diagnoses; (2) a cumulative diagnostic score [18], awarding two points for correct diagnoses, one point for plausible diagnoses, and zero points for incorrect diagnoses; (3) diagnostic confidence, rated on a visual analog scale from 0 to 10; and (4) case completion time, measured in seconds.

### Diagnostic accuracy evaluation

Diagnostic suggestions were independently rated as correct, plausible, or incorrect by two blinded board-certified rheumatologists. In cases of disagreement, a final rating was determined through discussion. The interrater agreement, using Cohen κ, was substantial (κ = 0.79).

### Other measures

Additionally, we analyzed the proportion of cases in which a correct or at least plausible diagnosis appeared among the top-ranked or top five suggested diagnoses.

To evaluate the relationship between diagnostic accuracy and diagnostic confidence, we assessed participants’ calibration using the over–under (O-U) index. Originally developed in psychology [19] and subsequently applied in internal medicine [2], the O-U index quantifies the direction and extent of miscalibration. It is computed as the difference between mean confidence and mean accuracy, yielding values from − 1 (complete underconfidence) to + 1 (complete overconfidence), with 0 indicating perfect calibration [19].

In the intervention group, we also evaluated AI over-reliance and under-reliance for the top diagnosis [20]. Over-reliance was defined as the proportion of instances in which participants accepted incorrect AI advice and consequently made an incorrect final decision, relative to all cases in which the AI advice was incorrect. Under-reliance was defined as the proportion of instances in which participants rejected correct AI advice and consequently made an incorrect final decision, relative to all cases in which the AI advice was correct.

Intervention group participants completed four items from the Telehealth Usability and Perceived Usefulness Short Questionnaire (TUUSQ) [21] after LLM usage: future intention to use, ease of use, user interface quality, and reliability. These items were rated on Likert scales ranging from 1 (strongly disagree) to 7 (strongly agree). The proportions of participants who agreed and disagreed with each statement were calculated.

### Statistical analysis

The study adhered to the CONSORT-AI Extension guideline. The required sample size of 68 participants was determined a priori, based on an assumed moderate effect size (Cohen’s d = 0.7), a two-sided significance level (α = 0.05), and 80% power. Analyses were conducted on the intention-to-treat (ITT) population, which included all randomized participants. Only available data were included in the analyses; no imputation was performed for missing values.

The effect of LLM support on the chance of selecting the correct top diagnosis (primary outcome) was evaluated using a generalized linear mixed model (GLMM) for binary outcomes with a logit link function. The model included random intercepts for participants (in order to adjust for the three vignettes per participant), along with fixed effects for group (intervention vs. control), gender, and age. The re-transformed effect estimate for the group variable is reported as adjusted Odds-ratio with corresponding 95% confidence interval (CI) as main confirmatory result.

For the secondary outcome (correct top diagnosis among the top 5 suggestions) and the exploratory analysis (correct or at least plausible diagnosis among the top or top 5 suggestions), we used a similar generalized linear mixed model (GLMM) as applied for the primary outcome. Group differences in cumulative diagnostic scores were assessed using a Welch t test, while group differences in diagnostic confidence and case completion time were compared using standard two-sample t tests.

Within-group comparisons in the intervention group (before vs. after use of the LLM) were analyzed using the same GLMM approach as for the primary outcome. Additional GLMM analyses included diagnostic confidence as an interaction term, with results reported as separate coefficients by group (for the primary outcome) or by time point (for within-group comparisons).

Statistical significance was defined as a 2-sided P value < 0.05. All statistical analyses were performed using R, version 4.3.1 (R Foundation for Statistical Computing), including the lme4 package for GLMM analyses.

## Results

### Participant characteristics

**Fig. 1:**
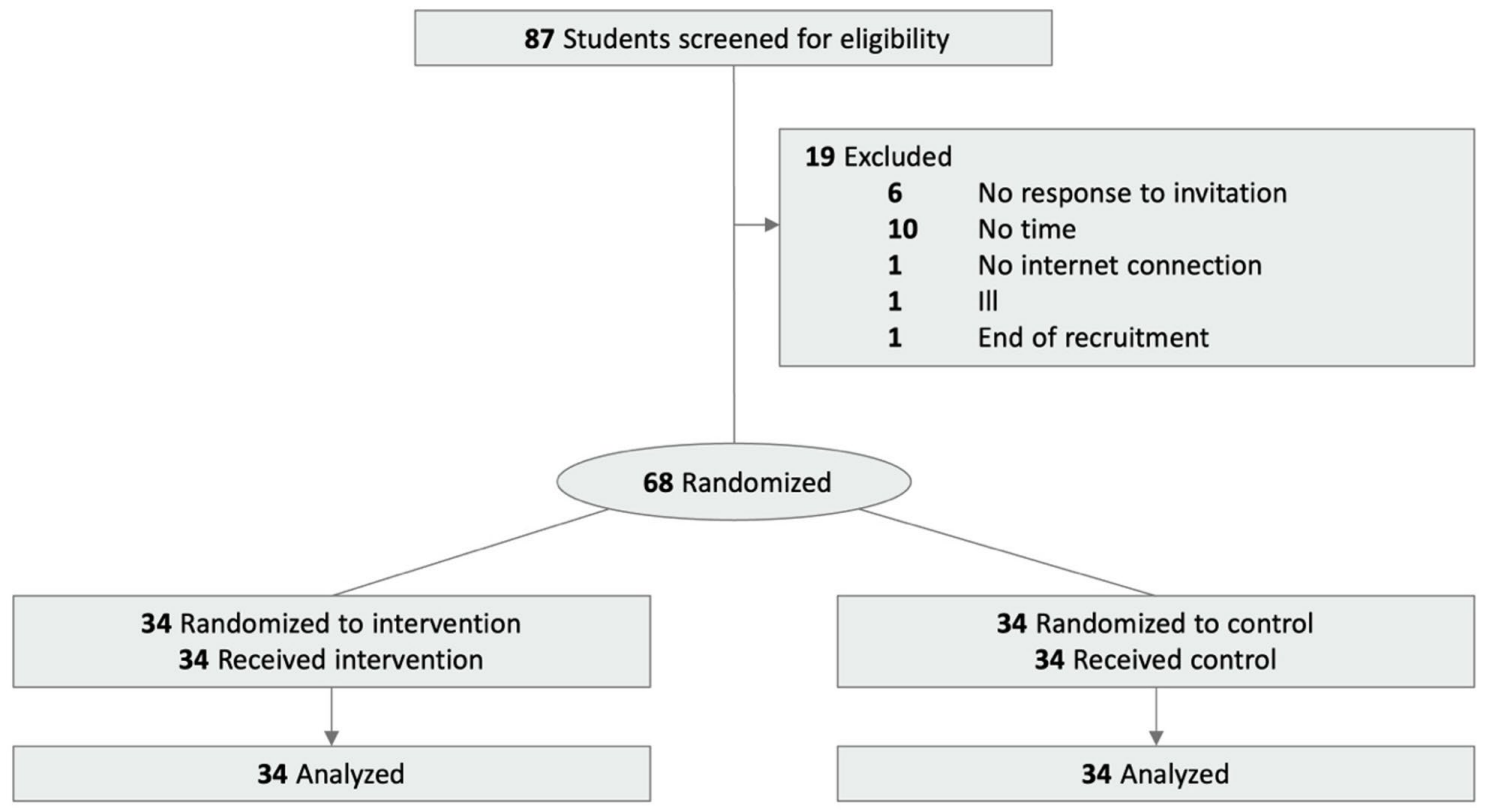
Trial participant flow diagram

Of 87 individuals screened, 68 were randomized (Fig. 1) and provided data for the primary analyses based on the intention-to-treat population.

Demographic characteristics (Table 1) were generally comparable between the two groups, with the majority of individuals having previously used LLMs (95.6%). The majority of participants self-reported their gender as female (61.8%), mean age (SD) was 24.8 (2.6) years.

**Table 1:** Baseline characteristics (intention-to-treat sample)

Characteristic	Participants, No. (%)		
	Total (*N* = 68)	Intervention group	Control group
		(*n* = 34)	(*n* = 34)
Age, mean (SD), y	24.8 (2.6)	24.8 (2.6)	24.8 (2.6)
Semester, median (IQR)	8.0 (7.0–8.0)	7.5 (7.0–8.0)	8.0 (7.3-8.0)
Gender, n (%)			
Female	42 (61.8)	23 (67.6)	19 (55.9)
Male	26 (38.2)	11 (32.4)	15 (44.1)
Previous usage of large-language-models, n (%)	65 (95.6)	34 (100.0)	31 (91.2)

### Primary outcomes

**Fig. 2:**
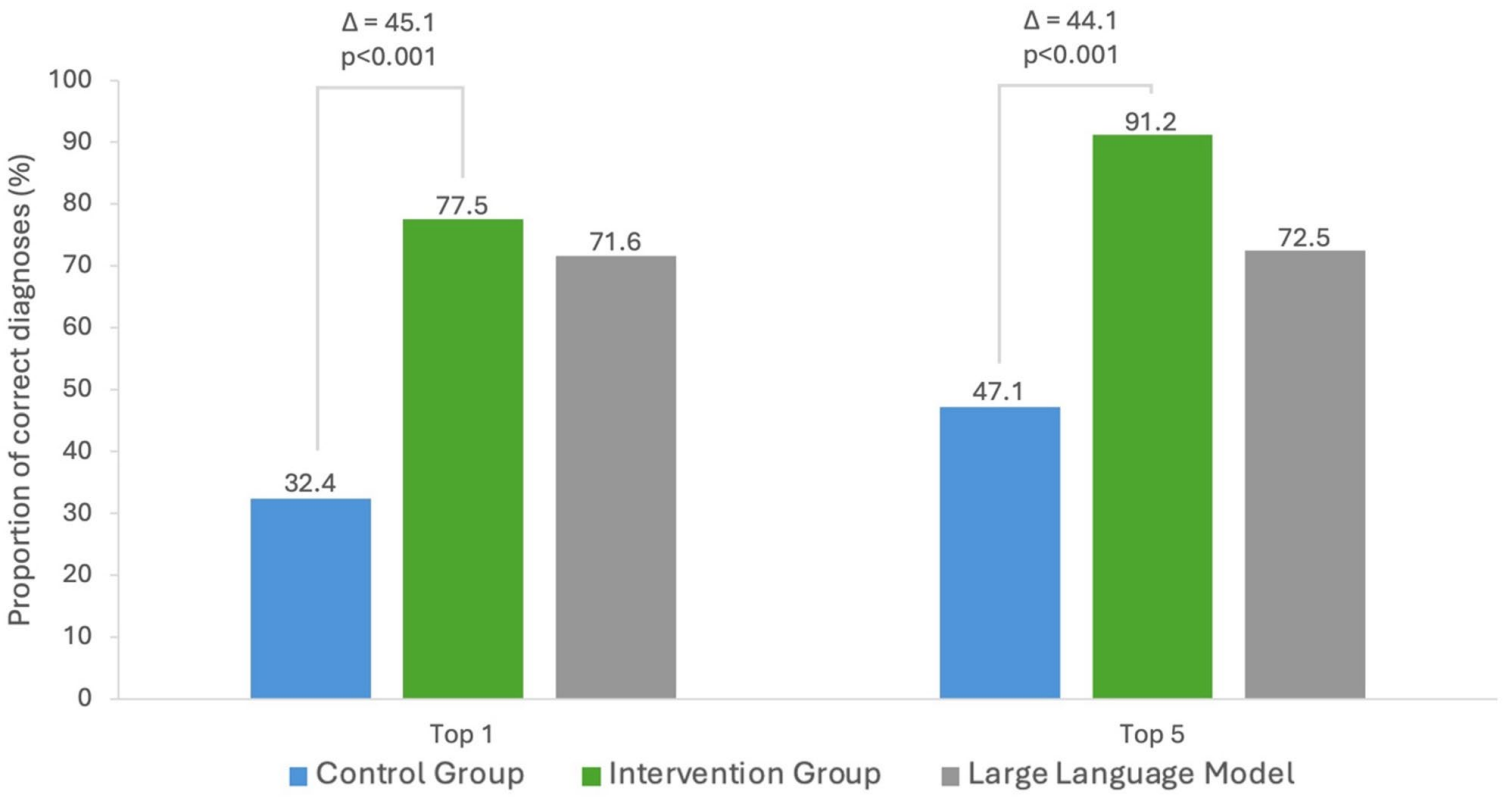
Proportion of correct diagnoses

Participants in the intervention group were significantly more likely to identify the correct top diagnosis compared with those in the control group (77.5% vs. 32.4%), yielding an adjusted odds ratio of 7.0 (95% CI, 3.8–14.4; *P*<.001) (Fig. 2).

This effect was particularly pronounced in case 1 (granulomatosis with polyangiitis), where 85.3% of LLM users selected the correct top diagnosis compared with 0% in the control group. Similarly, in case 3 (systemic lupus erythematosus), correct top diagnosis rates were higher in the LLM group (77.5% vs. 32.4%). In contrast, for case 2 (rheumatoid arthritis), the control group slightly outperformed the LLM group (55.9% vs. 52.9%) (see Supplementary Material 3). Case 2 described a 20 year old woman with symptoms for 4 months and the correct diagnosis was rheumatoid arthritis. 73.5% of participants in the intervention group correctly identified rheumatoid arthritis as the top diagnosis prior to using the LLM. The LLM itself provided the correct diagnosis for case 2 in only 17.6% of cases and frequently provided the wrong top diagnosis suggestion, juvenile idiopathic arthritis (67.6%). Notably, this incorrect suggestion influenced participants: although juvenile idiopathic arthritis was chosen as the top diagnosis by only one intervention participant before LLM use and by no control group participants, it appeared in 38.2% of top diagnoses after LLM assistance. This finding highlights the risk that users adopted incorrect suggestions provided by the model.

**Fig. 3:**
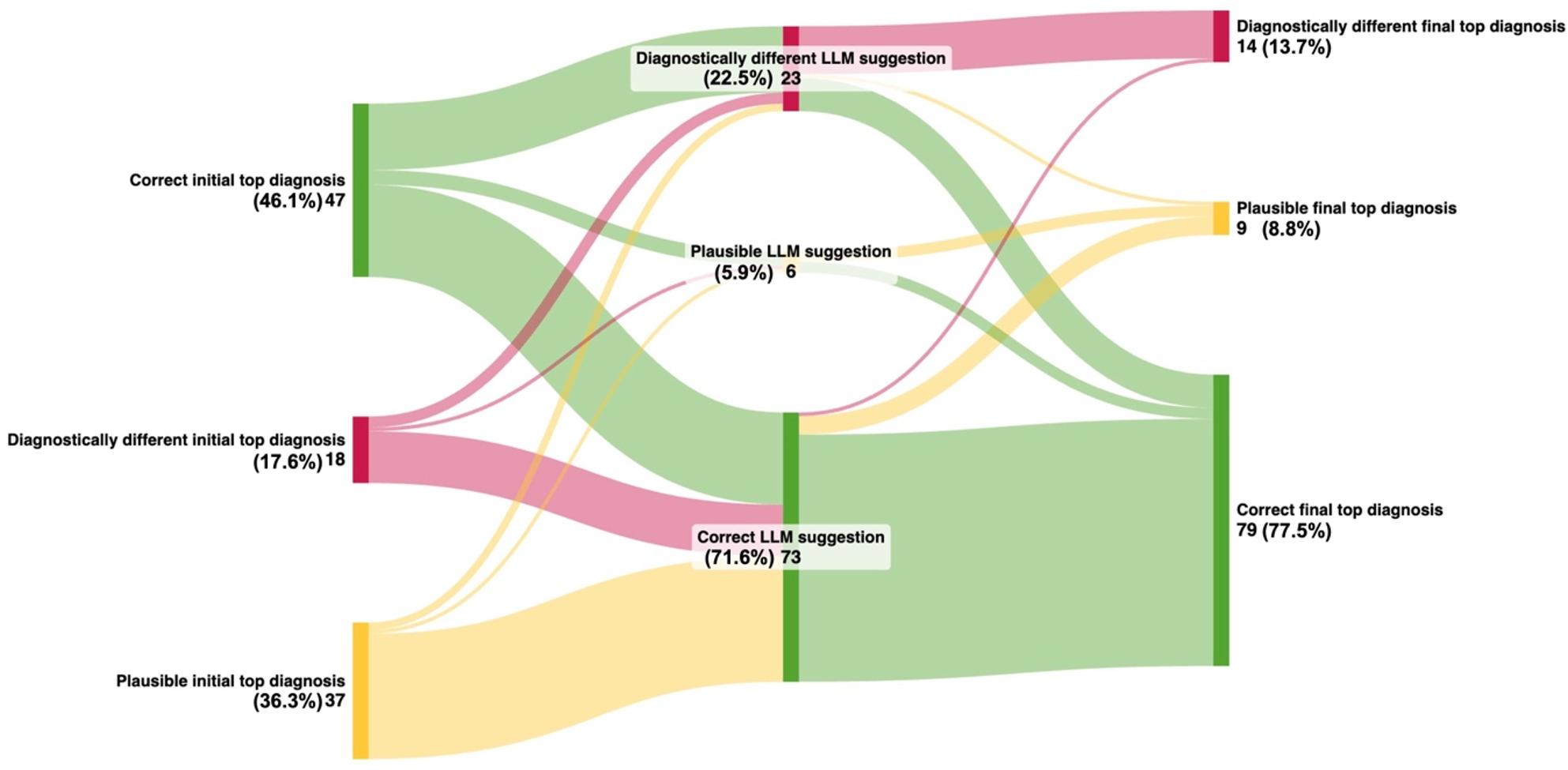
Sankey diagram displaying effect of LLM assistance on top diagnosis in the intervention group

Notably, participants in the intervention group also outperformed the LLM alone, with higher rates of correct diagnoses among the top suggestion (77.5% vs. 71.6%) and within the top five suggestions (91.2% vs. 72.5%), see Fig. 2. A within-group analysis comparing the intervention group before and after LLM use showed that participants were approximately four times more likely to identify the correct top diagnosis after using the LLM (adjusted odds ratio: 4.4; 95% CI: [2.3–8.5], *P*<.001). Figure 3 shows the impact of LLM assistance on the accuracy of top diagnoses within the intervention group.

### Secondary outcomes

Consistent with the primary outcome results, participants in the intervention group were significantly more likely to identify the correct diagnosis among top 5 suggestions compared with those in the control group (91.2% vs. 47.1%, *P*<.001), yielding an adjusted odds ratio of 11.8 (95% CI: [5.6–27.7], *P*<.001), see Fig. 2.

Mean cumulative diagnostic scores were significantly higher in the LLM group (mean [SD], 12.3 [2.3]) compared with the control group (6.7 [3.2]; Welch t₆₀.₂₂ = 8.1; *P*<.001). Diagnostic confidence in the IG increased significantly from before LLM use (mean 5.2 [SD 1.5] to after LLM use (mean 7 [SD 1.3], *P*<.001) and was significantly higher than in the control group (mean 6.1 [SD 1.2]; *P*<.001). Case completion time was significantly longer in the LLM group (mean 505 s [SD 131]) compared to the control group (mean 287 s [SD 106]; *P*<.001).

### Other outcomes

As additional resources, no participant used printed material and nearly all participants (98.5%) used Google Search. Additionally, 61.8% of participants (intervention group: 24 of 34; control group: 18 of 34) accessed Amboss (www.amboss.com*)*, and 10.3% (intervention group: 3 of 34; control group: 4 of 34) used Via Medici (www.viamedici.thieme.de)*.*

Half of the participants in the intervention group (50.0%) asked LLM follow-up questions, with a mean of 0.7 questions per participant (SD 0.8) (see Supplementary Appendix 4). For example, two participants who initially selected RA correctly as the top diagnosis for case 2, received JIA as the top suggestion from the LLM. They questioned this suggestion by asking the LLM about the age limit for JIA and ultimately chose to keep their original and correct RA diagnosis.

Participants in the intervention group were more likely to identify the correct or a plausible diagnosis as their top suggestion compared with the control group (86.3% vs. 77.5%) with an adjusted odds ratio of 2.6 (95% CI: [ 1.3–5.4], *P*=.009) and more frequently included a correct or plausible diagnosis among their top five suggestions (100.0% vs. 86.3%; *P*<.001) (see Supplementary Appendix 3). An adjusted odds ratio could not be calculated due to 100% accuracy in the intervention group and P values were obtained using nonparametric methods (Fisher exact and Wilcoxon tests yielded similar results).

Diagnostic calibration, measured by the O-U index, was 0.28 in the control group, indicating slight overconfidence (see Supplementary Appendix 5). In the intervention group, calibration was nearly perfect before using the LLM, with an O-U index of 0.06 (see Supplementary Appendix 5). After LLM use, calibration shifted toward underconfidence, with an O-U index of -0.07 (see Supplementary Appendix 5).

Similarly, when confidence was added to the generalized linear mixed model (GLMM) comparing the control group with the LLM-supported intervention group, we found that confidence was linked to diagnostic accuracy only in the control group. In the control group, higher confidence predicted more correct diagnoses (β = 0.87; 95% CI, 0.48–1.40; *P*<.001), but this was not the case in the intervention group (β = 0.03; 95% CI, − 0.26 to 0.31; *P*=.817). We saw the same pattern when comparing the intervention group before and after using the LLM. Confidence was associated with correct diagnoses before LLM use (β = 0.46; 95% CI, 0.22–0.76; *P*<.001), but not after (β = 0.03; 95% CI, − 0.61 to 0.70; *P*=.816). In the intervention group, AI over-reliance ratio was 0.28 (8/29) and under-reliance was 0.11 (8/73).

A total of 97.1% of participants in the intervention group stated they would use the LLM again for diagnostic support. Additionally, 91.2% found it easy to use, 94.1% considered the user interface to be well designed, and 67.6% agreed that they could quickly and easily correct any mistakes made during its use.

## Discussion

### Principal findings

This randomized clinical trial demonstrated that LLMs can effectively support medical students in diagnosing rheumatic diseases. Students supported by the LLM achieved greater diagnostic accuracy than both students working without LLM assistance and the LLM operating independently.

To our knowledge, the AIDRARER trial is the first randomized controlled trial to systematically evaluate the effectiveness of LLMs as diagnostic aids for medical students in rheumatology [14]. Although diagnosis is the most commonly explored application of LLMs, previous studies have primarily focused on benchmarking model performance using medical exam questions rather than assessing real-time use by healthcare professionals [14]. This trial responds to the growing call for studies involving actual user interaction [14]. Notably, this study also addressed an evidence gap by evaluating diagnostic confidence, an outcome that was among the least frequently assessed in a recent review of studies on LLMs in medicine [14].

Importantly, students who received LLM support outperformed both the LLM operating independently and students working without LLM assistance. This finding, though rarely demonstrated empirically [22], reinforces theoretical predictions that human–AI collaboration can achieve superior outcomes compared to either humans or AI working alone [23]. A previous study by McDuff et al. found that using the AIME medical LLM improved diagnostic accuracy among clinicians, however standalone LLM performance remained superior to clinician performance [24]. A landmark trial by Liu et al. reported mixed results with physician use of a fine-tuned LLM. While pulmonologists did not outperform the model even with its support, physicians with intermediate experience in endocrinology exceeded its performance [25]. A previous study demonstrated that LLMs did not improve clinical decision-making among laypersons, identifying incomplete data entry and suboptimal prompting as key limiting factors [26]. In our study, the use of a standardized prompt and clear instruction to input complete case data likely contributed to better outcomes. Participants also reported that the structured display of diagnostic probabilities and the option to ask follow-up questions improved their reasoning and decision-making. In some cases, follow-up questioning helped participants reject incorrect LLM suggestions (see Supplementary Appendix 4).

Additionally, our findings align with those of a recent benchmarking study [9] demonstrating that LLMs are significantly more accurate and faster than traditional diagnostic support systems, including certified medical products such as Ada. In a prior study using the same clinical vignettes [6], we evaluated Ada and found that it did not significantly improve the diagnostic accuracy of medical students. Furthermore, user acceptance was lower, with only 61% of medical students indicating they would use Ada again, compared to 97.1% for the LLM in the present trial.

These results further raise important ethical considerations regarding the use of certified but less effective tools versus non-certified yet higher-performing systems. Further regulatory guidance is needed to address the balance between certification standards and clinical utility in AI-assisted diagnostics [27]. Ideally, DDSS would integrate with electronic health records, lowering data entry barriers and enabling real-time decision support. Real-world implementation recently resulted in 16% fewer diagnostic errors in a large scale primary care study from Kenya [28].

Our finding of high acceptance mirrors prior DDSS research, where limited experience, low confidence, and case complexity were key drivers of adoption [29]. Yet younger and less experienced learners are also more prone to automation bias and reduced critical thinking [30]. This highlights that a primary objective of integrating AI into medical education should be the deliberate cultivation of critical thinking [11]. Hands-on, case-based training can provide valuable practice, but it must be complemented by structured approaches that encourage reflection, critical evaluation, and transparent reasoning. The recently proposed DEFT-AI framework [11] offers educators practical guidance on how to systematically embed AI into curricula while supporting these skills. In this study, follow-up questions proved valuable in uncovering incorrect disease suggestions. We recommend encouraging users to pose at least one challenging follow-up when uncertainty arises, and highlight the systematic evaluation of this strategy as a promising direction for future research.

Beyond critical thinking, a second key educational aim is to improve calibration between confidence and accuracy. Although LLM use increased both accuracy and confidence, it also introduced “mis-skilling,” with a shift toward underconfidence. Continous hands-on LLM practice with feedback may help students better align certainty with performance, though achieving lasting calibration remains difficult [31].

### Limitations

This study has several limitations. First, the findings are based on only three rheumatologic cases and a sample of medical students from a single institution. These cases had characteristic constellations and may overestimate performance relative to ambiguous real-world presentations. Additionally, we did not assess participants’ digital literacy, and a selection bias toward students with greater interest in digital health and LLMs cannot be excluded. Furthermore, students who participated earlier in the project may have shared or discussed the vignettes with peers who enrolled later in the study. Larger studies involving diverse clinical cases, real-world patient scenarios, and health care professionals with varying levels of experience are needed to validate and extend these findings. Second, LLM performance varied across cases, raising concerns about consistency. This limitation might be addressed in future research by combining the outputs of multiple models through weighted ensemble approaches [23]. Third, although study personnel ensured that all case information was entered, the frequency of initially incomplete or incorrect data entry was not recorded. Fourth, while we employed a standardized prompt, this approach may have led to an overestimation of performance compared to the more variable and often ambiguous prompting strategies used in real-world practice. Given the substantial influence of prompting on outcomes, medical education programs should incorporate prompt design and refinement as a core competency to support effective and responsible use of LLMs [11]. Fifth, it remains unknown whether the vignettes were included in the LLM’s training data. Sixth, although ChatGPT-4o was selected due to its widespread use, its cloud-based and proprietary nature precludes input of sensitive patient data. Further research is needed to evaluate promising open-source and locally deployable models [9, 32]. Seventh, the absence of qualitative feedback limited a more detailed exploration of participants’ reasoning and experiences.

## Conclusions

This randomized clinical trial demonstrated that large language models can support medical students in diagnostic reasoning. Students assisted by the LLM achieved higher diagnostic accuracy than both the LLM alone and students without LLM assistance, highlighting the value of human-AI collaboration in clinical reasoning. These findings suggest that LLMs hold promise as diagnostic aids, though further studies are needed to confirm their impact across settings and on clinical outcomes.

## Supplementary Information

Supplementary Material 1.

Supplementary Material 2.

Supplementary Material 3.

Supplementary Material 4.

Supplementary Material 5.

## Data Availability

The raw data supporting the conclusions of this article will be made available by the authors upon reasonable request.

## Abbreviations

AI: Artificial Intelligence
CI: Confidence Interval
CONSORT: Consolidated Standards of Reporting Trials
DDSS: Diagnostic Decision Support System
GLMM: Generalized Linear Mixed Model
ITT: Intention-To-Treat
JIA: Juvenile Idiopathic Arthritis
LLM: Large Language Model
RA: Rheumatoid Arthritis
SD: Standard Deviation
SLE: Systemic Lupus Erythematosus
TUUSQ: Telehealth Usability and Perceived Usefulness Short Questionnaire
