# Supplementary figures and images for: Large language models enhance diagnostic reasoning of medical students in rheumatology: a randomized controlled trial
Source: BMC Med Educ. 2026 Mar 25;26:579. doi: 10.1186/s12909-026-09079-w (PMC13064386; doi:10.1186/s12909-026-09079-w)

**Initial standardized LLM prompt**

**
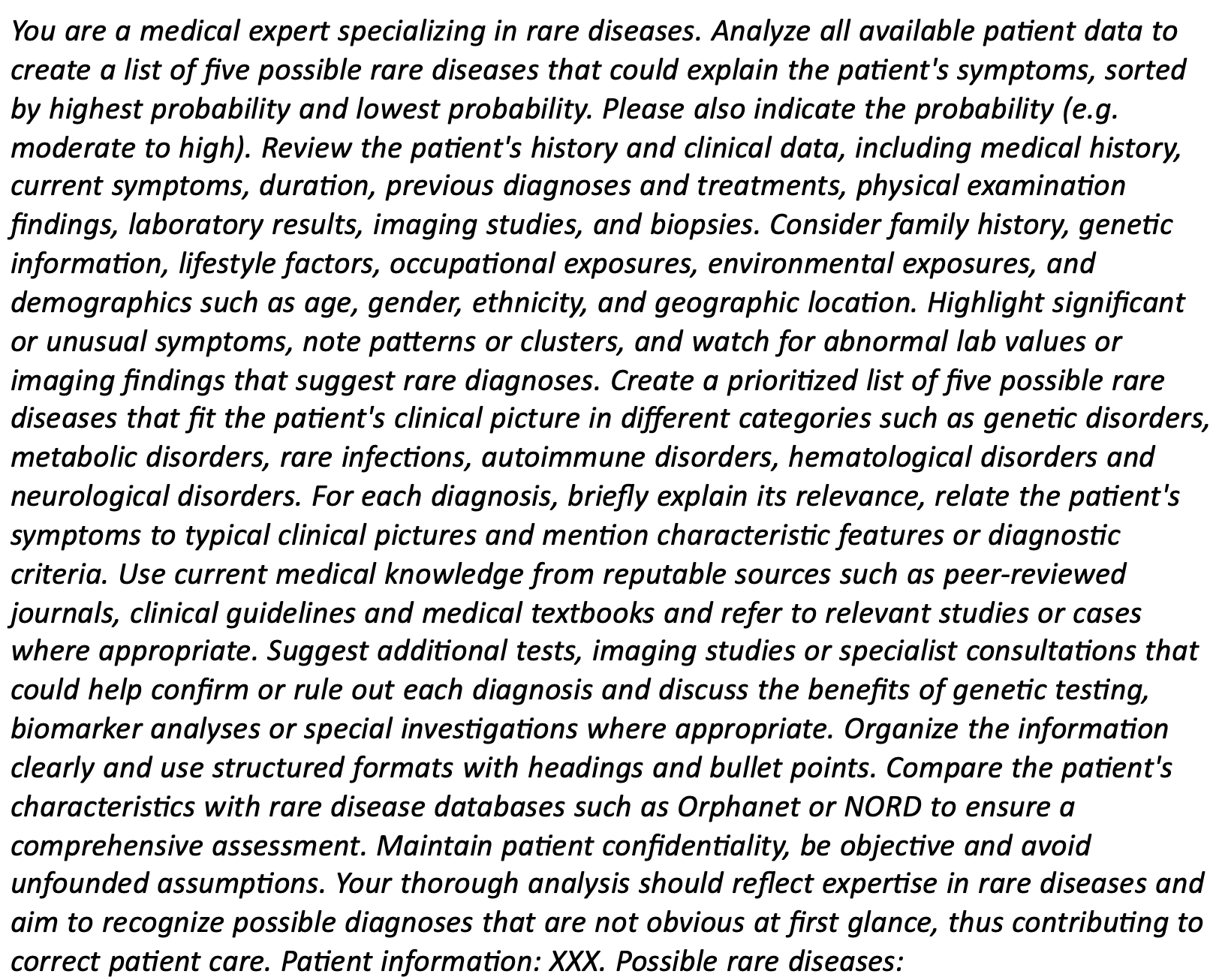
**

Supplement: Supplementary file 1 — Supplementary Material 1. [file 12909_2026_9079_MOESM1_ESM.docx]
