# Supplementary material for: Large language models enhance diagnostic reasoning of medical students in rheumatology: a randomized controlled trial
Source: BMC Med Educ. 2026 Mar 25;26:579. doi: 10.1186/s12909-026-09079-w (PMC13064386; doi:10.1186/s12909-026-09079-w)
Supplement: Supplementary file 2 — Supplementary Material 2. [file 12909_2026_9079_MOESM2_ESM.docx]

**Example of LLM response for case 1**

Based on the provided clinical presentation and history of the 43-year-old male patient, including:

- **Systemic symptoms**: prolonged fever, malaise, fatigue
- **ENT symptoms**: recurrent sinusitis, nasal congestion, ear infections, epistaxis
- **Pulmonary symptoms**: cough with hemoptysis, mild dyspnoea
- **Musculoskeletal**: migratory arthralgia
- **Ophthalmic**: painful red eye (suggestive of episcleritis or uveitis)

This constellation of findings strongly suggests a **systemic inflammatory or autoimmune disorder**, possibly with **vasculitic involvement**. Below is a prioritized differential diagnosis list of rare diseases, considering categories like autoimmune, infectious, and genetic disorders:

**1. Granulomatosis with polyangiitis (GPA, formerly Wegener’s granulomatosis)**

**Probability: High**

**Rationale**: GPA is a small-to-medium vessel vasculitis characterized by necrotizing granulomas involving the upper and lower respiratory tracts and kidneys.

- Fits well with **recurrent sinusitis, otitis, epistaxis**, and **pulmonary symptoms** including hemoptysis.
- **Migratory arthralgia** and **ocular involvement** (e.g., scleritis) are common.
- **Systemic symptoms** such as malaise and fatigue further support the diagnosis.

**Characteristic features**:

- Positive **PR3-ANCA (c-ANCA)** in ~90% of generalized cases.
- Chest imaging: **nodules**, **cavities**, **infiltrates**.
- Renal involvement may appear later (e.g., hematuria, rising creatinine).

**Recommended tests**:

- **c-ANCA (PR3)**, **p-ANCA (MPO)**.
- **Chest CT**, **urinalysis**, **creatinine**.
- Biopsy of sinus or lung lesion showing granulomatous inflammation with vasculitis.

**References**:

- Yates M, et al. Ann Rheum Dis. 2016; EULAR recommendations for ANCA-associated vasculitis.
- Orphanet entry: ORPHA73235

**2. Eosinophilic Granulomatosis with Polyangiitis (EGPA, formerly Churg-Strauss)**

**Probability: Moderate**

**Rationale**: This is another ANCA-associated vasculitis, typically affecting people with asthma and eosinophilia. However, ANCA-positive variants can present without prominent eosinophilia or asthma.

- ENT symptoms and **pulmonary involvement** with systemic signs may be present.
- **Migratory arthralgia** and **ocular involvement** can occur.

**Features**:

- Peripheral eosinophilia, asthma, mononeuritis multiplex.
- Less likely due to **absence of asthma** and eosinophilia in the given case.

**Tests**:

- CBC with differential (for eosinophilia).
- p-ANCA (MPO), nerve conduction studies (if neuropathy suspected).
- Sinus/lung biopsy if needed.

**References**:

- Jennette JC et al., Arthritis Rheum. 2013.
- Orphanet entry: ORPHA98246

**3. Relapsing Polychondritis**

**Probability: Moderate to low**

**Rationale**: A rare autoimmune disease that primarily affects cartilaginous structures (e.g., ears, nose, trachea), often presenting with **auricular chondritis, sinusitis, arthralgia**, and **ocular inflammation**.

- This patient’s **ear infections**, **nasal congestion**, and **arthralgia** may suggest early or atypical presentation.
- Tracheal involvement can lead to **cough and dyspnoea**.

**Features**:

- Red, tender ears sparing the lobule.
- Associated with other autoimmune conditions or vasculitis.

**Tests**:

- Clinical diagnosis supported by imaging of trachea (CT).
- Biopsy of auricular cartilage if needed.

**References**:

- Orphanet entry: ORPHA830

**4. Sarcoidosis**

**Probability: Low to moderate**

**Rationale**: Multisystem granulomatous disease with **pulmonary**, **ENT**, **ocular**, and **musculoskeletal** involvement.

- Can cause **chronic cough**, **sinusitis**, **red eye (uveitis)**, and **arthralgia**.
- Hemoptysis is **less typical**, and granulomatous inflammation may resemble GPA.

**Features**:

- Bilateral hilar lymphadenopathy on chest imaging.
- Non-caseating granulomas on biopsy.
- Serum ACE and calcium may be elevated.

**Tests**:

- Chest CT, serum ACE, calcium, biopsy of accessible lesion.
- Ophthalmologic examination for uveitis.

**References**:

- Baughman RP et al., N Engl J Med. 2001.
- Orphanet entry: ORPHA797

**5. Goodpasture’s Syndrome (Anti-GBM Disease)**

**Probability: Low**

**Rationale**: Pulmonary-renal syndrome characterized by **alveolar hemorrhage (hemoptysis)** and **rapidly progressive glomerulonephritis**.

- Could explain **hemoptysis and fatigue**, but **ENT symptoms**, **ocular involvement**, and **joint complaints** are atypical.

**Features**:

- Hemoptysis, dyspnoea, anemia, hematuria.
- Positive **anti-GBM antibodies**.

**Tests**:

- Anti-GBM antibody, urinalysis, creatinine.
- Chest imaging, renal biopsy (linear IgG deposition).

**References**:

- Hellmark T et al., J Autoimmun. 2014.
- Orphanet entry: ORPHA
