# Supplementary material for: Large language models enhance diagnostic reasoning of medical students in rheumatology: a randomized controlled trial
Source: BMC Med Educ. 2026 Mar 25;26:579. doi: 10.1186/s12909-026-09079-w (PMC13064386; doi:10.1186/s12909-026-09079-w)

Proportion of correct diagnoses as the top suggestion (Top 1) and within the top 5 suggestions (Top 5) as by case and study group

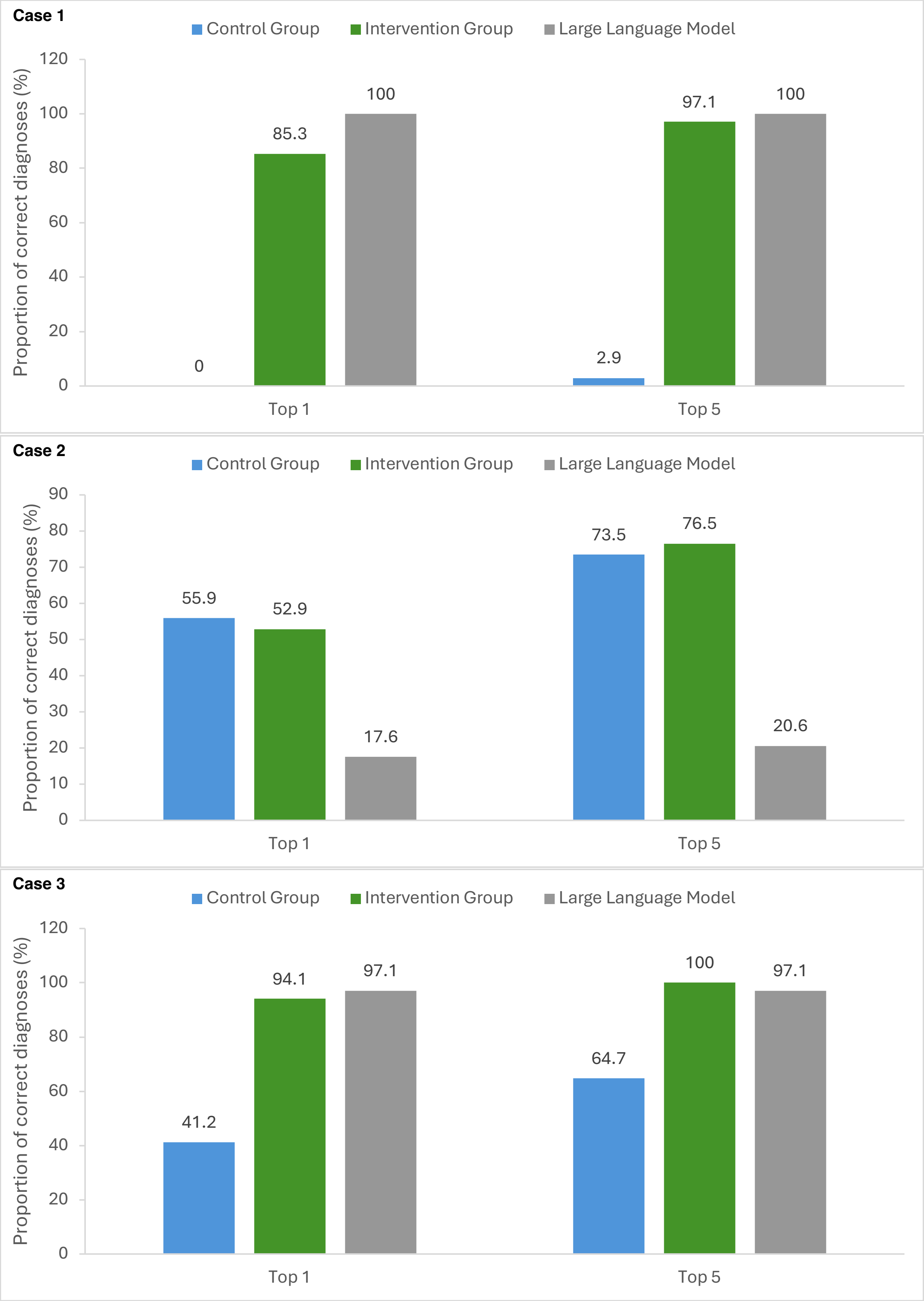

Supplement: Supplementary file 3 — Supplementary Material 3. [file 12909_2026_9079_MOESM3_ESM.pdf]
