# Supplementary material for: Large language models enhance diagnostic reasoning of medical students in rheumatology: a randomized controlled trial
Source: BMC Med Educ. 2026 Mar 25;26:579. doi: 10.1186/s12909-026-09079-w (PMC13064386; doi:10.1186/s12909-026-09079-w)
Supplement: Supplementary file 4 — Supplementary Material 4. [file 12909_2026_9079_MOESM4_ESM.docx]

**Follow-up prompts for the three cases**

| **Case 1** | How does probability come about; to what extent a disease that is not rare is perhaps even more probable? |
| --- | --- |
|  | At what age are the diagnoses likely? |
|  | How common are the diseases in the general population? |
|  | Why do you think GPA? |
|  | Would you rule out general illnesses such as pneumonia? |
|  | How sure are you about your five suspected diagnoses? |
|  | Which of the five suspected diagnoses is the most common? |
|  | Why is the PCD out of the question? |
|  | How confident are you in your choice of diagnoses and the ranking? |
|  | How likely is a tumor of the NNH? |
| **Case 2** | At what age are the diagnoses likely? |
|  | Why would you rule out rheumatoid arthritis? |
|  | How sure are you about your five suspected diagnoses? |
|  | What are the early symptoms of SLE and what is the age of first manifestation? |
|  | What is the age of onset of JIA? |
|  | Does JIA occur more frequently in female patients than in male patients? |
|  | Can JIA also be diagnosed in adulthood? |
|  | What is the age limit for the JIA? |
|  | Why is JIA listed as the top diagnosis and not RA or viral arthritis? |
|  | Why are RA, reactive arthritis and ankylosing spondylitis not on the list? |
|  | How likely is RA or osteoarthritis? |
| **Case 3** | How likely is a PAN? |
|  | What is the Shwal sign? |
|  | At what age is dermatomyositis most common?; At what age does reactive arthritis occur? |
|  | How likely are the diagnoses compared to yours? Reactive arthritis, fibromyalgia, psoriatic arthritis, Lyme disease |
