# Supplementary material for: Large language models enhance diagnostic reasoning of medical students in rheumatology: a randomized controlled trial
Source: BMC Med Educ. 2026 Mar 25;26:579. doi: 10.1186/s12909-026-09079-w (PMC13064386; doi:10.1186/s12909-026-09079-w)
Supplement: Supplementary file 5 — Supplementary Material 5. [file 12909_2026_9079_MOESM5_ESM.docx]

**Supplementary file 5**

Calibration diagrams displaying the proportion of correct top diagnoses at each confidence level in the control group and intervention group

**Figure 1**

Calibration diagram displaying the proportion of correct top diagnoses at each confidence level in the control group


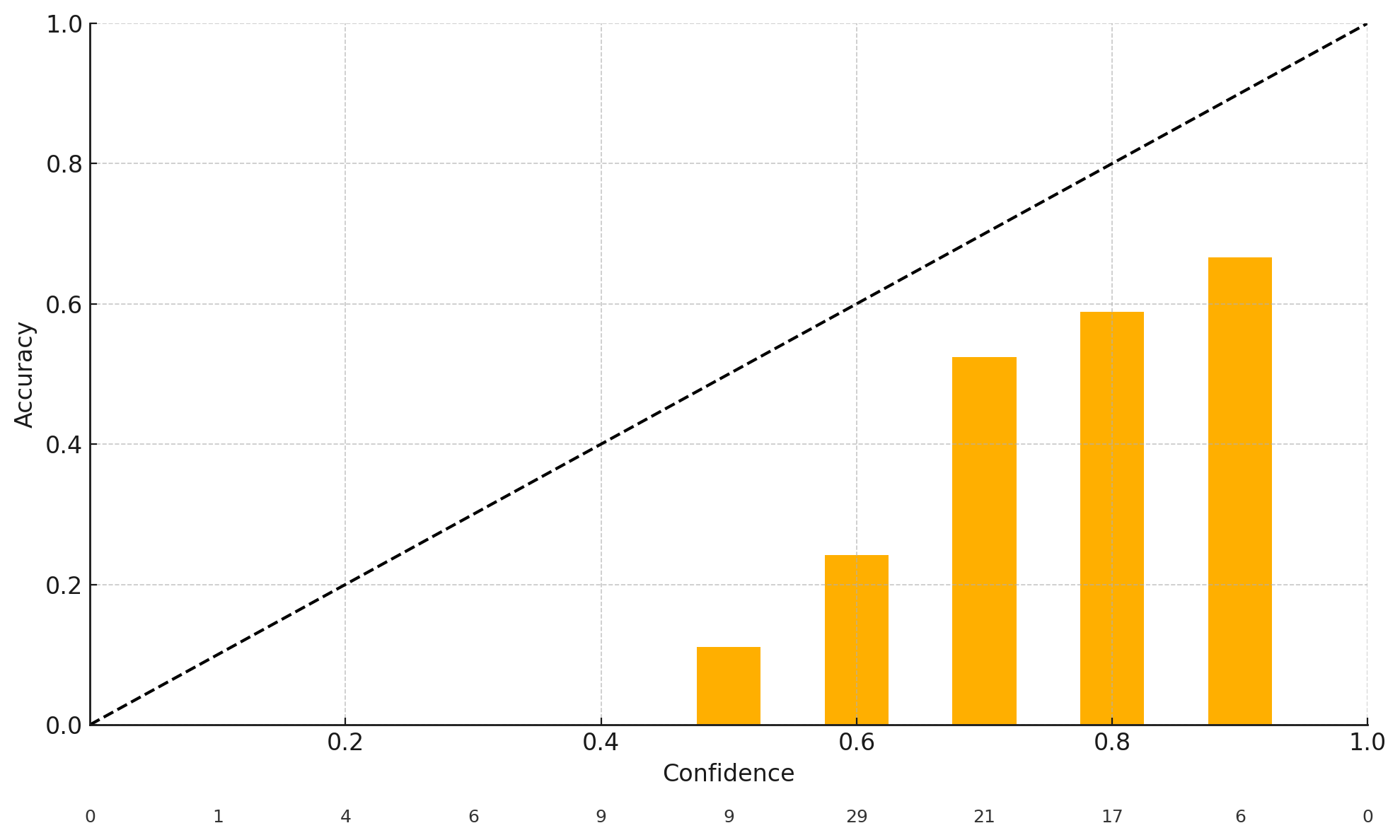


**Figure 2**

Calibration diagram displaying the proportion of correct top diagnoses at each confidence level in the intervention group before LLM use.


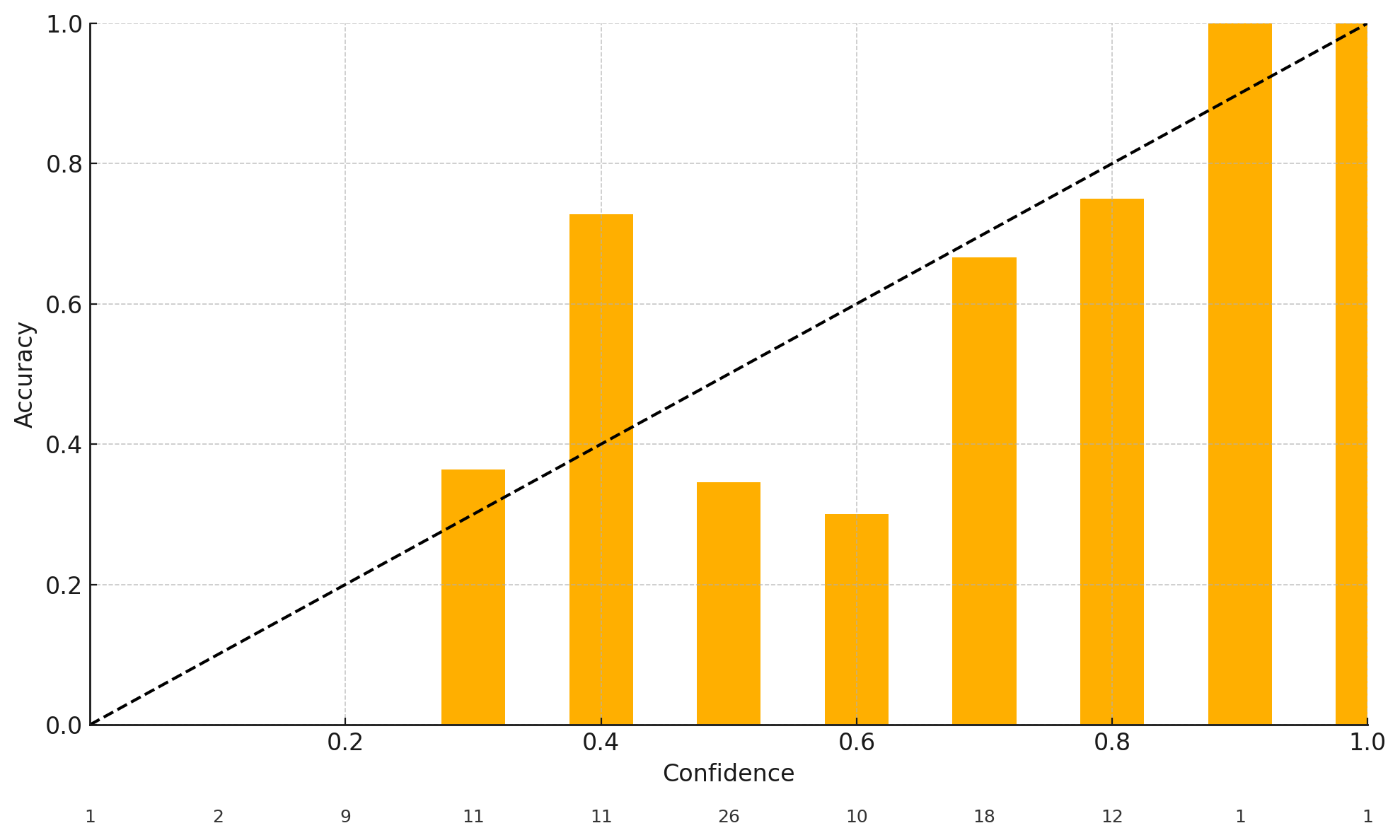


**Figure 3**

Calibration diagram displaying the proportion of correct top diagnoses at each confidence level in the intervention group after LLM use.

**
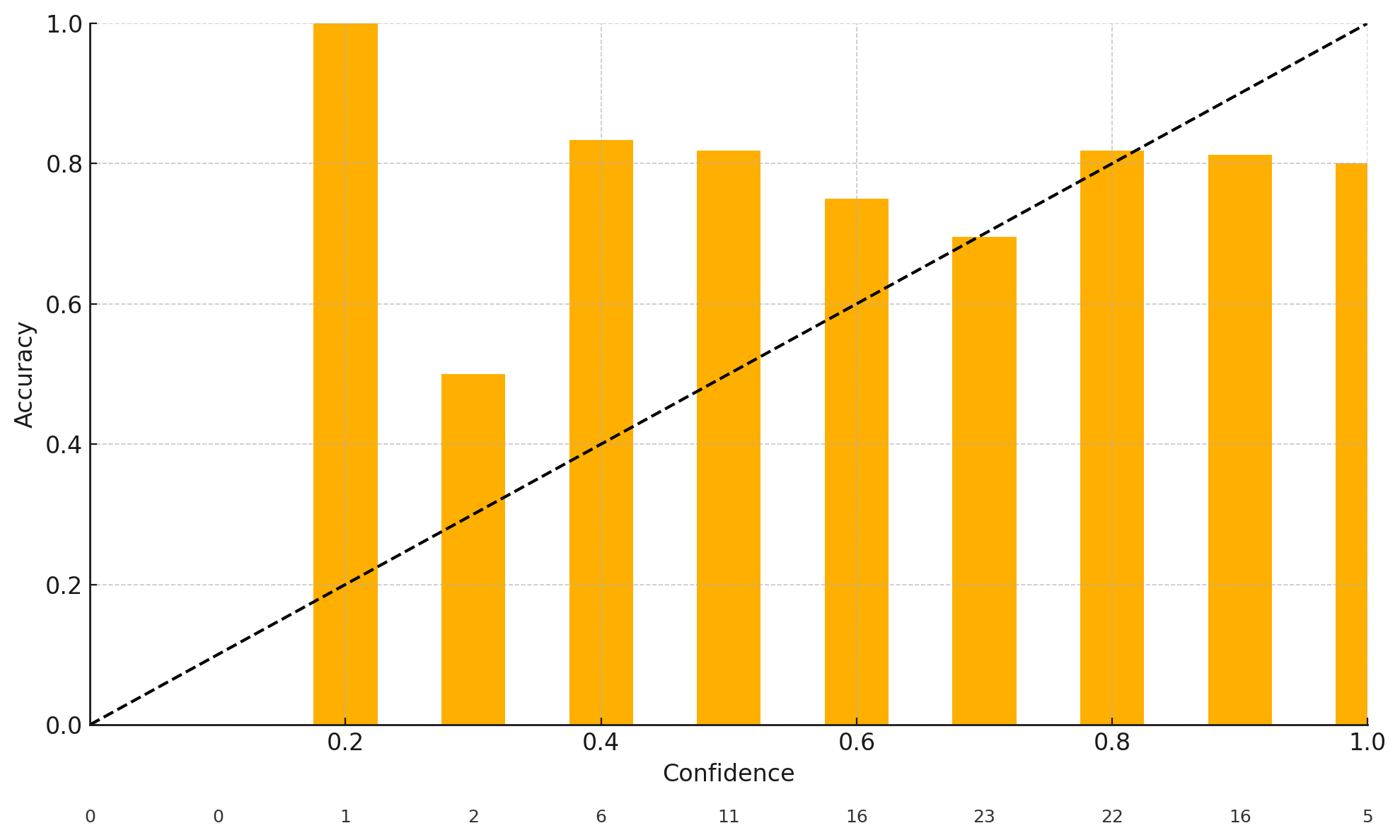
**
